# 609 The State of Burn Care Training During General Surgery Residency

**DOI:** 10.1093/jbcr/irae036.327

**Published:** 2024-04-17

**Authors:** Sebastian Q Vrouwe, Brittany Davis, Alexandra M Lacey

**Affiliations:** University of Chicago, Chicago, IL; Regions Burn Unit, Saint Paul, MN; University of Chicago, Chicago, IL; Regions Burn Unit, Saint Paul, MN; University of Chicago, Chicago, IL; Regions Burn Unit, Saint Paul, MN

## Abstract

**Introduction:**

There is an ongoing and looming shortage of burn surgeons. Workforce reports suggest possible hurdles attracting general surgeons into burn care include the removal of the general surgery training requirement of a formal burn rotation over a decade ago. The purpose of this study was to (1) determine the current state of burn care in general surgery residency and (2) identify what barriers might exist for general surgeons pursuing a practice that involves burn care.

**Methods:**

Surveys were electronically distributed to General surgery program directors and residents, respectively, during the 2022–2023 academic year.

**Results:**

A total of 82 programs (response, 13%) and 62 program directors (17%) agreed to send the surveys on to their residents. Of the participating programs, 48 had one or more residents complete a survey (59%), with a total of 250 general surgery residents (10%) participating. Burn care was felt to be an important component of training by most program directors (82%) and residents (81%). The majority of responding program directors included a burn unit rotation (61%), and the majority of responding residents had or would complete a burn rotation in their residency (59%). Of those residents who had completed a burn rotation, 33% felt it increased their interest, 44% felt it had no impact on their interest, and 24% felt it decreased their interest. Of the residents that did not have a formal burn rotation, 61% were interested in participating in one. Regardless of whether the residents had exposure to burn care, 50% of surveyed resident participants expressed interest in a career that involved some degree of burn care. The top factors that would discourage a trainee from practicing burn care in the future included the narrow scope of practice (44%), nature of burn operations (43%), nature of burn care (39%), and insufficient exposure during their training (32%).

**Conclusions:**

This study indicates that burn rotations in general surgery training are important in establishing interest in burn care. Insufficient exposure to burn care may be one of the top factors discouraging trainees from a career in burn surgery.

**Applicability of Research to Practice:**

In light of the projected workforce shortage, these findings may encourage programs to incorporate a regular burn rotation and increase exposure to a career in burn care for general surgery residents who would otherwise not pursue the field.

